# SARS-CoV-2 Spike protein promotes vWF secretion and thrombosis via endothelial cytoskeleton-associated protein 4 (CKAP4)

**DOI:** 10.1038/s41392-022-01183-9

**Published:** 2022-09-22

**Authors:** Kan Li, Liu Yao, Jin Wang, Hao Song, Yan-hong Zhang, Xue Bai, Kai Zhang, Dong-ming Zhou, Ding Ai, Yi Zhu

**Affiliations:** 1 The Province and Ministry Co-sponsored Collaborative Innovation Center for Medical Epigenetics, Department of Physiology and Pathophysiology, Tianjin Medical University, Tianjin, China; 2 Department of Biochemistry and Molecular Biology, Tianjin Medical University, Tianjin, China; 3 Department of Pathogen Biology, Tianjin Medical University, Tianjin, China

**Keywords:** Cell biology, Pathogenesis

**Dear Editor**,

Although progressive respiratory failure is the primary cause of death in patients with COVID-19, thromboembolic complications further increased the mortality rates. Endothelial dysfunction, a pro-inflammatory and pro-coagulant state characterized by increased interaction with leukocytes and platelets, is reported to play a key role in COVID-19-associated thrombosis;^1^ however, its underlying mechanism remains unclear.

von Willebrand factor (vWF) is primarily secreted by endothelial cells (ECs) and functions as a transporter of pro-coagulant factor VIII (FVIII). It is also an initiator of platelet adhesion and aggregation, leading to thrombus formation. A clinical study reported that ICU patients with COVID-19 had a significantly higher level of vWF in blood than non-ICU patients,^2^ suggesting a role of vWF in COVID-19-associated coagulopathy. To examine the effects of vWF regulation in COVID-19-associated thrombosis, we used the recombinant purified Spike protein of severe acute respiratory syndrome coronavirus 2 (SARS-CoV-2) to mimic SARS-CoV-2 invasion. Spike protein had no effects on vWF expression but induced vWF secretion in a dose- and time-dependent manner in human umbilical vein ECs (Supplementary Fig. 1a, b; Fig. 1a, b). Simultaneously, FVIII-vWF binding and platelet adhesion to ECs were significantly elevated after Spike protein stimulation (Fig. 1c–e). These data suggest a potential role of vWF secretion in COVID-19-associated coagulopathy.

**Fig. 1:**
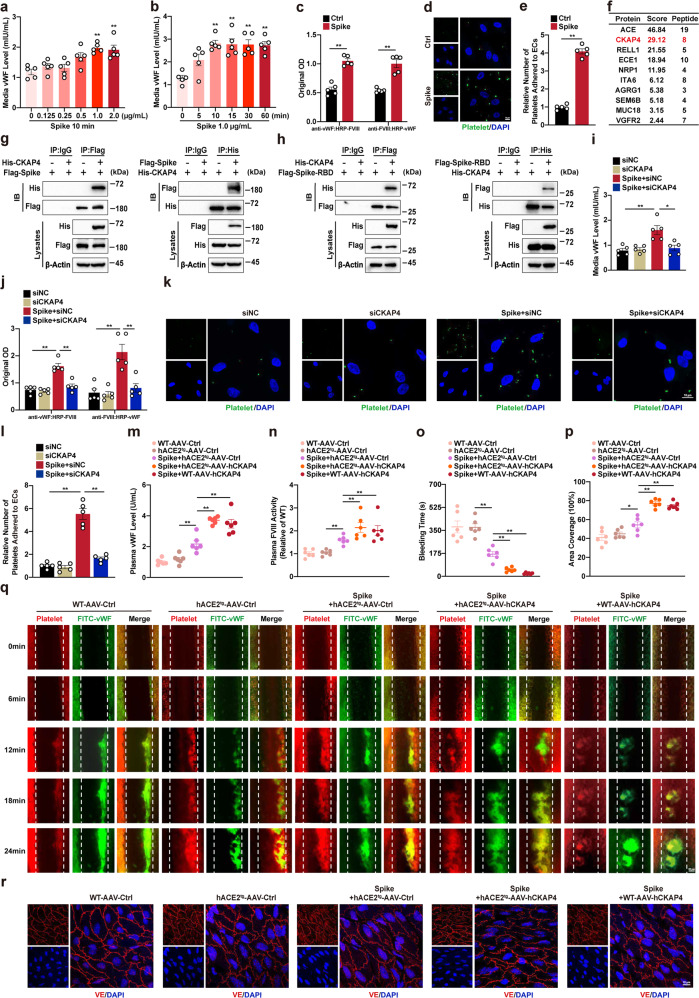
SARS-CoV-2 Spike protein binds to endothelial CKAP4 to promote vWF secretion and thrombosis in vitro and in vivo. **a**, **b** Cell culture media was collected from human umbilical vein ECs (HUVECs) after Spike protein treatment. vWF levels in the culture media of HUVECs treated with different doses of Spike protein treatment for 10 min (**a**) or with the same dose of Spike protein (1.0 μg/mL) for different time periods (**b**). One way ANOVA, *n* = 5, **P < 0.01 vs Ctrl. **c** FVIII-vWF binding assays analyzed the binding of vWF secreted by HUVECs to exogenous recombinant human FVIII protein. Unpaired two-tail *t* test, *n* = 5, **P < 0.01. **d**, **e** Rat platelets were isolated and labeled with calcein-AM fluorescent probes and incubated with Spike protein-treated HUVECs. Representative images of calcein-AM fluorescence staining (scale bar, 10 μm) (d) and quantification of number of platelets adhered to HUVECs (e). Platelet (green) and DAPI (blue). Unpaired two-tail *t* test, *n* = 5, **P < 0.01. **f** Mass spectrometry identified strongly interacted membrane receptors with Spike protein; the scores and peptides of top 10 candidates are shown. Plasmid of His-CKAP4 was co-transfected with Flag-Spike protein plasmid (**g**) or Flag-Spike protein-receptor-binding domain (RBD) plasmid (**h**) in HEK293T cells for 36 h. Whole-cell lysates were immunoprecipitated and then immunoblotted with antibodies against the indicated proteins. **i**–**l** HUVECs were treated with Spike protein for 10 min after transfection of CKAP4-siRNA (siCKAP4) or negative control (siNC) for 36 h. **i** vWF levels in the culture media; Two-way ANOVA, *n* = 5, *P < 0.05; **P < 0.01. **j** FVIII-vWF binding assay; Two-way ANOVA, *n* = 5, **P < 0.01. **k**, **l** Rat platelets were isolated and labeled with calcein-AM fluorescent probes and incubated with Spike protein-treated HUVECs. Representative images of calcein-AM fluorescence staining (scale bar, 10 μm) (**k**) and quantification of number of platelets adhered to HUVECs (**l**). Platelet (green) and DAPI (blue). Two-way ANOVA, *n* = 5, **P < 0.01. Six-week-old male hACE2^tg^ and C57BL/6 WT mice were injected with AAV-hCKAP4 or AAV-Ctrl for 2 weeks, and then treated with Spike protein for 10 min. **m** Plasma vWF levels; **n** Plasma FVIII activity; **o** Tail bleeding times. **p** Quantification of (**q**) area coverage of thrombus in mesenteric vessels at 24 min. **q** Thrombus formation in mesenteric vessels of mice at different time points via live cell workstation (scale bar, 50 μm). Rhodamine-labeled platelets together with FITC-conjugated anti-vWF antibody showed thrombus formation in mesenteric vessels. Two-way ANOVA, *n* = 6 mice in each group. *P < 0.05; **P < 0.01. **r** Representative images of VE-cadherin-labeling of ECs membrane. *n* = 6 mice in each group, VE-cadhe**r**in (red) and DAPI (blue), (scale bar, 10 μm)

Endothelial membrane receptors are vital regulators of vWF secretion. To explore the potential receptors involved in Spike protein-induced vWF secretion, endothelial membrane proteins were extracted and incubated with His-tagged Spike protein, a bait protein that had been immobilized to cobalt chelate affinity resin. By utilizing the poly-His pull-down assay, proteins bound to Spike protein were obtained and analyzed using proteomics. The top 10 potential candidates are listed in Fig. 1f, excluding angiotensin-converting enzyme-2 (ACE2). ACE2 is a well-known receptor for SARS-CoV-2 infection; however, treatment of ECs with DX600, a potent and selective peptide inhibitor of ACE2, showed no repression of Spike protein-induced vWF secretion (Supplementary Fig. 1c, d), implying an indirect role of ACE2 in vWF secretion. Although ACE was the first candidate binding with Spike protein (Supplementary Fig. 1e), knockdown of ACE did not block Spike protein-induced vWF secretion (Supplementary Fig. 1f). Here, we find cytoskeleton-associated protein 4 (CKAP4) is a novel receptor for Spike protein invasion, as evidenced by its specific interaction with Spike protein and receptor-binding domain of Spike protein (Fig. 1g, h). CKAP4 is widely expressed in various tissues and cells and acts as a ligand-specific membrane receptor. The report has shown elevation of CKAP4 membrane translocation in ECs weakens the integrity of endothelial adherence junction; however, little is known about the role of endothelial CKAP4 in coagulopathy. We found silencing endothelial CKAP4 abolished Spike protein-induced vWF secretion, FVIII-vWF binding, and platelet adhesion to ECs (Supplementary Fig. 1g; Fig. 1i–l). Moreover, the role of CKAP4 in coagulopathy was further confirmed in human lung microvascular ECs (Supplementary Fig. 1h–k). Thus, CKAP4 is a novel receptor for Spike protein recognition and binding, and essential for Spike protein-induced coagulopathy.

To determine the availability and function of CKAP4, we constructed adeno-associated virus-human CKAP4 (AAV-hCKAP4) and AAV-Control (AAV-Ctrl) under the Tie1 promoter. 5 × 10^11^ vector genomes AAV-hCKAP4 or equivalent dose of AAV-Ctrl were injected to transgenic mice that express human ACE2 (hACE2^tg^) and C57BL/6 WT mice via the tail vein to obtain endothelial-specific hACE2^tg^-AAV-hCKAP4 and WT-AAV-hCKAP4 mice, respectively. Immunofluorescence staining revealed that hCKAP4 expression was distinctly augmented in endothelium of mesenteric arteries of both types of AAV-hCKAP4 mice (Supplementary Fig. 1l, m). To further explore the pathophysiological relevance of CKAP4 with Spike protein-related thrombosis, mice were injected with or without Spike protein (80 μg/kg) via the tail vein for 10 min. Plasma vWF levels exhibited no changes between hACE2^tg^ and WT mice, and a slight increase was observed in hACE2^tg^ mice injected with Spike protein, however, the highest plasma vWF levels were observed in mice with hCKAP4 overexpression after Spike protein injection, irrespective of hACE2^tg^ (Fig. 1m). Plasma vWF often circulates in combination with FVIII to regulate platelet aggregation and clot formation. Plasma FVIII activity showed a uniform trend with plasma levels of vWF (Fig. 1n). Bleeding time reflects platelet function and clot-forming ability of the body. The shortest periods of bleeding were observed consistently in both types of hCKAP4 overexpression mice with Spike protein administration (Fig. 1o). We then monitored the dynamic changes of vWF secretion and thrombus formation in mesenteric vessels of mice using live cell workstation. Mice were pretreated with fluorescein isothiocyanate (FITC)-labeled vWF antibody and rhodamine-labeled platelets before Spike protein stimulation. FITC-labeled vWF secretion and rhodamine-labeled platelet counts were significantly increased at the sites of thrombus formation in mesenteric vessels of both strains of mice with hCKAP4 overexpression induced by FeCl_3_ after Spike protein injection (Fig. 1p, q). In addition, EC-specific overexpression of CKAP4 did not affect EC structure in mice with or without Spike protein-stimulation (Fig. 1r). Therefore, Spike protein-caused coagulopathy were primarily attributed to its binding with CKAP4 and downstream vWF secretion, rather than impairing EC structure.

Several clinical studies have reported endotheliitis^3^ and severe endothelial injury, including thrombosis^4^ occurred in patients who died from COVID-19 infection, suggesting endothelial injury played an important role in COVID-19. High plasma vWF levels were associated with the severity of COVID-19. This raises the possibility that vWF secretion is required to facilitate Spike protein-induced thrombosis. The current study demonstrated that Spike protein induced vWF secretion and coagulopathy, and provided new evidence for COVID-19-associated coagulopathy from the perspective of endothelial dysfunction. Although report supports ACE2 is a primary receptor for SARS-CoV-2 entry, ACE2 showed low or absent expression in human ECs.^5^ Additionally, no interaction was detected between ACE2 and Spike protein by our membrane proteomics and no repression of Spike protein-induced vWF secretion by DX600 further confirmed other potential receptor might mediate Spike protein-associated thrombosis. Excitingly, our study verified that endothelial CKAP4, as a novel receptor for Spike protein, predominantly potentiates Spike protein-induced vWF secretion and thrombosis. The current data provide a new target and therapeutic approach to treat patients with SARS-CoV-2-associated thrombotic complications.

## Supplementary information

Supplemental material

## Data Availability

Proteomics raw data have been deposited to the ProteomeXchange Consortium via the iProX partner repository with the dataset identifier PXD036314.
