# Supplementary material for: SARS-CoV-2 Spike protein promotes vWF secretion and thrombosis via endothelial cytoskeleton-associated protein 4 (CKAP4)
Source: Signal Transduct Target Ther. 2022 Sep 22;7:332. doi: 10.1038/s41392-022-01183-9 (PMC9500075; doi:10.1038/s41392-022-01183-9)
Supplement: Supplementary file 1 — Supplemental material [file 41392_2022_1183_MOESM1_ESM.docx]

**Supplementary materials for**

**SARS-CoV-2 Spike protein promotes vWF secretion and thrombosis via binding to endothelial cytoskeleton associated protein 4**

Kan Li^1#^, Liu Yao ^1#^, Jin Wang^1^, Hao Song^1^, Yan-hong Zhang^1^, Xue Bai^2^, Kai Zhang^2^, Dong-ming Zhou^3^, Ding Ai^1*^, and Yi Zhu^1*^

Correspondence: Ding Ai ([dingai@tmu.edu.cn](mailto:dingai@tmu.edu.cn)) or Yi Zhu ([zhuyi@tmu.edu.cn](mailto:zhuyi@tmu.edu.cn))

**This PDF file includes:**

Materials and methods

Supplementary figure 1

Original and uncropped films of western blots

**Materials and methods**

**Reagents**

Spike protein (40589-V08B1), Flag-Spike protein-receptor binding domain (VG40592-CF), Myc-ACE2 (HG10108-CM), Myc-ACE (HG11598-CM), His-CKAP4 (HG18475-CH), and anti-HRP-vWF (10973-R111-H) and anti-HRP-FVIII (13909-R402-H) antibodies were procured from Sino Biological (Beijing, China). Antibodies against vWF (ab154193), CKAP4 (ab152154), and CD31 (ab24590) and vWF enzyme-linked immunosorbent assay (ELISA; ab208980, ab108918) and FVIII activity assay kits (ab204696) were procured from Abcam (Cambridge, MA, USA). Antibodies against Flag-Tag (14793), Myc-Tag (2276), and His-Tag (12698) were procured from Cell Signaling Technology (Danvers, MA, USA); anti-CKAP4 (sc-393544) and anti-vWF (sc-53466) antibodies were procured from Santa Cruz Biotech (Santa Cruz, CA, USA); β-Actin (66009) was procured from Proteintech (Rosemont, IL, USA); and the ACE2 inhibitor DX600 (S9666) was procured from Selleck (Houston, TX, USA). CKAP4 siRNA (sc-95758) and control siRNA (sc-37007) were obtained from Santa Cruz Biotech. Mem-per™ plus membrane protein extraction kit (89842), pierce pull-down poly-His protein: protein interaction kit (21277), Alexa Fluor 488- and 594-conjugated secondary antibodies, calcein-AM (C3100MP), and FITC antibody labeling kit (53027) were procured from Thermo Fisher Scientific (Grand Island, NY, USA). Rhodamine 6G (HY-D0309) was procured from Med Chem Express (Monmouth Junction, NJ, USA).

**Cell isolation and culture**

Primary human umbilical vein endothelial cells (HUVECs) were isolated and cultured in basal M199 medium (Gibco, Grand Island, NY, USA) supplemented with 10% fetal bovine serum (Gibco) in a humidified incubator with 5% CO_2_ at 37 °C.^1^ Cells at passages 4 to 6 were used for the experiments after starvation with 1% bovine serum albumin (BSA) for 4 h.

HEK293T cells were cultured in Dulbecco's modified Eagle medium (Life-iLab, Shanghai, China) supplemented with 10% fetal bovine serum in humidified atmosphere of 5% CO_2_ at 37 °C. Cells at 70–80% confluency were used for the experiments.

**ELISA**

For determining vWF levels in the culture medium, HUVECs were seeded in 48-well plates, and the medium was replaced when 70–80% confluency was reached. HUVECs were treated with different concentrations of Spike protein for different time periods after 4 h serum-starvation, then the culture medium was collected for vWF ELISA detection. For determining vWF levels in plasma, 50 μL of peripheral blood was collected from the tail vein of mice of different groups. The blood samples were mixed with 10 μL of 3.2% sodium citrate and centrifuged at 200 *g* for 10 min; the supernatant was collected for vWF ELISA detection. The concentration of vWF in the culture medium and plasma were determined using a commercial ELISA kit (Abcam), according to the manufacturer's instructions.

**Platelet adhesion**

Blood samples were collected form the apex of the rat heart and diluted with equal volume of tissue diluent (Solarbio, Beijing, China). The mixture was centrifuged at 200–250 *g* for 10 min; the supernatant was transferred to a fresh tube and washed with phosphate-buffered saline (PBS). After centrifugation at 500 *g* for 20 min, the residues (platelets) were collected and resuspended in 1 mL of Tyrode's buffer (Solarbio) for counting. Appropriate amounts of platelets were pipetted and labeled with calcein-AM (2 μM) in M199 media for 1 h at 37 °C. Then, 2 × 10^6^ platelets were added to each well of 6-well plate for platelet adhesion. Non-adhered platelets were washed and removed after 1 h, and the adhered platelets were fixed with HUVECs in paraformaldehyde solution 4% in PBS for 15 min at 25 °C. Images were acquired on a laser confocal ultra-high-resolution microscope 900 (Zeiss, Oberkochen, Germany) with a 63× oil objective lens.

**Plasmid construction and FVIII-vWF binding assay**

A FVIII fragment plasmid was generated by Generalbiol company (Anhui, China) by cloning parts of the FVIII (amino acids 1649–2332) using pGEX-6P-1 bacterial vector to obtain a recombinant FVIII protein. Two methods were used to evaluate the ability of FVIII binds with vWF, as described previously.^2^ In the first method, Immuno 96-well microtiter plates were coated with 0.5 μg/mL of anti-vWF antibody at 4 °C overnight. The plates were washed five times with TBST buffer (50 mmol/L Tris HCl, pH 7.6, 150 mmol/L NaCl, and 0.05% Tween 20) after each step and blocked with TBST containing 3% BSA for 2 h at 37 °C. Purified recombinant FVIII protein (1.0 μg) was preincubated with 100 μL of media containing vWF from ECs after different treatments and then added to the plates. After incubation for 2 h, the vWF/FVIII samples were added to the plates and incubated with anti-FVIII horseradish peroxidase-conjugated secondary antibodies. The FVIII bound to immobilized vWF was quantified by measuring the absorbance at 450 nm. In the second method, Immuno 96-well microtiter plates were coated with 0.5 μg/mL of anti-FVIII antibody overnight at 4 °C. The plates were subsequently washed and blocked, as described for the first method. The vWF/FVIII samples were added to the plates and incubated with anti-vWF horseradish peroxidase-conjugated secondary antibodies. The vWF bound to immobilized FVIII was quantified by measuring the absorbance at 450 nm.

**Membrane protein extraction and** **poly-His pull-down assay**

For membrane protein extraction, cells were collected and suspended in a wash solution. After centrifugation at 300 *g* for 5 min, the cells were collected and then permeabilized with permeation buffer at 4 °C for 10 min with gentle rocking. The permeated cells were centrifuged at 16,000 *g* for 15 min, and the supernatant containing cytoplasmic proteins was transferred to a fresh centrifuge tube. Then, 0.5 mL of solubilizing buffer was added to the tube, and the precipitates were resuspended at 4 ℃ for 30 min with continuous mixing. The mixture was centrifuged at 16,000 *g* for 15 min; the supernatant containing soluble membrane proteins was collected and used for subsequent experiments.

For poly-His pull-down assay, the spin column containing HisPur cobalt resin was first equilibrated and immobilized with the poly-His-tagged bait protein, Spike protein, at 4 °C for 30 min with gentle rocking. The resin was washed five times at 1,250 *g* for 30 s. The above extracted membrane proteins were then incubated with the immobilized spin column containing poly-His-tagged Spike protein at 4 °C for 1 h with gentle rocking. Adequate elution buffer was added to the spin column, and prey proteins were collected and subjected to LC-MS/MS sequencing, and data analysis as described.^3^

**Transient transfection**

For gene knockdown experiments, HUVECs were transfected with siRNA-negative control (siNC), -CKAP4 (siCKAP4), or -ACE (siACE) for 36 h using lipofectamine RNAi MAX, followed by treatment with Spike protein (1.0 μg/mL) for 10 min. For transient transfection, HEK293T cells were co-transfected with the plasmids Flag-Spike protein and Myc-ACE2, or Flag-Spike protein and Myc-ACE, or Flag-Spike protein and His-CKAP4, or Flag-Spike protein-RBD and His-CKAP4 with the aid of lipofectamine 3000 in a 10-cm dish for 36 h. The culture medium from siRNA transfection HUVECs were used for vWF ELISA detection and cells from plasmids transfection were lysed for western blotting and co-immunoprecipitation assay. IgG as a negative control.

**Co-immunoprecipitation (Co-IP)**

Co-IP assay was performed according to a previously described procedure.^4^ Briefly, transfected HEK293T cells were washed with PBS and lysed with a non-denatured lysate (Solarbio) containing complete protease inhibitor cocktail and phosSTOP phosphatase inhibitor (Roche, Mannheim, Germany) for 30 min at 4 °C. The lysate was then centrifuged at 13,800 *g* for 15 min at 4 ºC. The supernatant was collected and subjected to protein quantification. Then, 1.0 μg protein was incubated with the corresponding label magnetic beads at 4 ºC overnight under constant rotation. After washing beads with NP-40 lysis buffer, the protein extract was collected and mixed with SDS loading buffer, boiled for 5 min, and subjected to immunoblotting.

**Western blotting**

Whole-cell or membrane proteins were extracted from cells using a commercial membrane extraction kit or RIPA buffer (Solarbio) containing complete protease inhibitor cocktail and phosSTOP phosphatase inhibitor (Roche, Mannheim, Germany). Proteins in the extract were separated using 8% or 10% SDS-PAGE and then transferred onto nitrocellulose membranes. Target bands on the membrane were incubated with corresponding primary antibodies at 4 °C overnight and subsequently with horseradish peroxidase-conjugated secondary antibodies (1:5000) for 1 h at 25 °C. Proteins were visualized using the ECL chemiluminescence system (Tanon, Shanghai, China). β-Actin served as an internal standardization control. For the IP assay, loading control was dependent on the target protein precipitated using IP antibody.

**Animal experiments**

Six-week-old transgenic mice that express human angiotensin-converting enzyme-2 (ACE2) (hACE2^tg^, NM-TG-200002; Shanghai Model Organisms Center, Inc. Shanghai, China) and C57BL/6 WT mice (Beijing Vital River Laboratory Animal Technology, Beijing, China) were housed in specific pathogen free conditions. They were injected with 5 × 10^11^ vector genomes AAV-hCKAP4 under the Tie1 promoter or equivalent dose of AAV-Ctrl via the tail vein, and then succumbed after 2 weeks. The animal study was reviewed and approved by the Committee of Tianjin Medical University on Animal Experimentation (TMUaMEC2022009).

**Immunofluorescence staining**

Mesenteric aortas from mice were washed, fixed, and incubated with blocking-permeabilization buffer (0.05% Triton X-100, 1% BSA) for 1 h. The aortas were incubated with the primary antibodies (anti-hCKAP4, the endothelial marker anti-CD31 and VE-cadherin) (1:100) at 4 °C overnight. Corresponding secondary antibodies (1:200) were added, and images were acquired using a Zeiss LSM 900 system.

**Tail bleeding time**

As a partial length of the tails of six-week-old hACE2^tg^ mice was clipped for genotyping before procuring, excessive distal segments of the tails of other mice were removed to ensure uniform length before performing experiments in the present study. After few days of healing, the mice were anesthetized with isoflurane (2–3%). A uniform distal 2 mm segment of the tail was removed and immediately placed in 0.9% NaCl and maintained at 37°C. The bleeding time was recorded from the time of damage until the cessation of blood. The assay was terminated at 10 min.

**Monitoring of thrombus formation**

Platelets were isolated from C57BL/6 WT mice and labeled with rhodamine. The FITC-conjugated vWF antibodies and rhodamine-labeled platelets were injected into mice via the tail vein, which followed by isolation of mesenteric blood vessels and incubation mesenteric blood vessels with 20% FeCl_3_. The real-time process of thrombus formation was monitored by live cell workstation (Westborough, MA, USA) as described by Li et al.^5^

**Statistical analysis**

Data are presented as mean ± SEM. GraphPad Prism v8.0 (GraphPad Software, San Diego, CA, USA) was used for all statistical analyses. Unpaired Student’s *t*-test (two-tailed) or one-way, two-way ANOVA were carried out with Tukey’s correction for multiple comparisons. *P* < 0.05 was considered statistically significant.

**REFERENCES**

1. Yao, L. et al. Regulation of YAP by mammalian target of rapamycin complex 1 in endothelial cells controls blood pressure through COX-2/mPGES-1/PGE2 cascade. *Hypertension*. **74,** 936-946 (2019).

2. Bendetowicz, A. V., Morris, J. A., Wise, R. J., Gilbert, G.E. & Kaufman, R.J. Binding of factor VIII to von willebrand factor is enabled by cleavage of the von Willebrand factor propeptide and enhanced by formation of disulfide-linked multimers. *Blood*. **92,** 529-538 (1998).

3. He, J. et al. Yes-associated protein promotes angiogenesis via signal transducer and activator of transcription 3 in endothelial cells. *Circ Res.* **122,** 591-605 (2018).

4. Liu, M. et al. Macrophage K63-linked ubiquitination of YAP promotes its nuclear localization and exacerbates atherosclerosis. *Cell Rep*. **32,** 107990 (2020).

5. Li, P. et al. Myosin IIa is critical for cAMP-mediated endothelial secretion of von Willebrand factor. *Blood*. **131**, 686-698 (2018).

**Supplemental figure 1.** **a, b** Western blot analysis and quantification of vWF expression in HUVECs treated with different concentrations of Spike protein for 10 min **(a)** or with the same concentration of Spike protein (1.0 μg/mL) for different time periods **(b)**. One way ANOVA, n = 5. **c** HEK293T cells were co-transfected with Flag-Spike protein and Myc-ACE2 plasmids for 36 h. Whole-cell lysates of HEK293T were immunoprecipitated and immunoblotted using antibodies against the indicated proteins. **d** vWF levels in the culture media of HUVECs treated with Spike protein for 10 min after administration with DX600 for 30 min. Two-way ANOVA, n = 5, ***P* < 0.01. **e** HEK293T cells were co-transfected with Flag-Spike protein and Myc-ACE plasmids for 36 h. Whole-cell lysates of HEK293T were immunoprecipitated and immunoblotted using antibodies against the indicated proteins. **f** vWF levels in the culture media of HUVECs treated with Spike protein for 10 min after siRNA transfection of ACE-siRNA (siACE) or negative control (siNC) for 36 h. Two-way ANOVA, n = 5, **P* < 0.05. **g** Western blot analysis of CKAP4 expression in HUVECs subjected to siRNA transfection of CKAP4-siRNA (siCKAP4) or negative control (siNC) for 36 h. Band visualization (top) and quantification (bottom) of CKAP4 expression. Unpaired two-tail *t* test, n = 5, ***P* < 0.01. **h-k** Human lung microvascular ECs (HULEC-5a) were treated with or without Spike protein for 10 min after transfection of CKAP4 -siRNA (siCKAP4) or negative control (siNC) for 36 h. **h** vWF levels in the culture media; **i** FVIII-vWF binding assay; **j, k** Rat platelets were isolated and labeled with calcein-AM fluorescent probes and incubated with Spike protein-treated HULEC-5a. Representative images of calcein-AM fluorescence staining (scale bar, 10 μm) (**j**) and quantification of number of platelets adhered to HULEC-5a (**k**). Platelet (green) and DAPI (blue). Two-way ANOVA, n = 5, ***P* < 0.01. **l, m** Six-week-old male hACE2^tg^ and C57BL/6 WT mice were injected with AAV-hCKAP4 or AAV-Ctrl for 2 weeks, and then treated with Spike protein for 10 min. Representative images of immunofluorescence staining **(l)** and quantification of **(m)** hCKAP4 in the mesenteric aortic endothelium (scale bar, 20 μm); hCKAP4 (green), CD31 (red), and DAPI (blue). Two-way ANOVA, n = 6, ***P* < 0.01.

**Original and uncropped films of western blots**
